# Differential Influences of Wind-Blown Sand Burial on Bacterial and Fungal Communities Inhabiting Biological Soil Crusts in a Temperate Desert, China

**DOI:** 10.3390/microorganisms10102010

**Published:** 2022-10-11

**Authors:** Rongliang Jia, Yanhong Gao, Lina Zhao, Tao Zhang, Hui Guo, Wanxue You, Yulong Duan

**Affiliations:** 1 Shapotou Desert Research and Experiment Station, Northwest Institute of Eco-Environment and Resources, Chinese Academy of Sciences, Lanzhou 730000, China; 2 College of Food and Bioengineering, Henan University of Science and Technology, Luoyang 471023, China; 3 Institute of Medicinal Biotechnology, Chinese Academy of Medical Sciences, Beijing 100050, China; 4 College of Resources and Environmental Sciences, Nanjing Agricultural University, Nanjing 210095, China; 5 Ningxia Shapotou National Nature Reserve Administration, Zhongwei 755007, China; 6 Naiman Desertification Research Station, Northwest Institute of Eco-Environment and Resources, Chinese Academy of Sciences, Tongliao 028300, China

**Keywords:** biological soil crusts (BSCs), sand burial, microbial community composition, bacteria, fungi, succession

## Abstract

Biological soil crusts (BSCs) are an integration of external photoautotrophs and internal heterotrophic communities. Sand burial is a ubiquitous disturbance that affects the biodiversity and ecological function of BSCs, but little is known about the influence of sand burial on microbial communities in arid sandy deserts. Here, based on a long-term field experiment and utilizing high-throughput sequencing, we assessed the influence of sand burial on bacterial and fungal communities inhabiting two typical successional stages of BSCs (cyanobacterial crusts for early successional stage and mixed crusts for late successional stage) at the three-sand buried depth (0, 0.5, and 10 mm) in the Tengger Desert, Northern China. We found that the diversity, abundance, and composition of the bacterial and fungal communities were all altered by the sand burial treatment. Different indicator taxa were identified in unburied and buried (shallow and deep) BSCs. Changes in soil properties caused by sand burial have been suggested as a possible cause of changes in the bacterial and fungal community composition in BSCs.

## 1. Introduction

Biological soil crusts (BSCs) are complex, intimate assemblies of photoautotrophs, cyanobacteria, algae, lichens, and mosses, and heterotrophic bacteria and fungi in different proportions [1,2,3]. In general, these photoautotrophs and heterotrophic communities spatially anchor the outer and inner assembly of the BSCs architecture in the uppermost soil surface of the drylands [4]. Furthermore, the dynamics of the external and internal sub-consortia collectively determine the multiple functions of BSCs, such as stabilization, nutrient cycling, and hydrological processes in desert ecosystems [5,6]. A growing body of literature indicates that BSCs could be influenced by climate change and anthropogenic disturbances [5,6,7] and then change the external and internal characteristics of BSCs and their ecological functions [3,8]. Although bacteria and fungi locate the inside matrix of BSCs [9,10], their communities are reported to be susceptible and respond differentially to multiple environmental change factors, such as soil and climatic aridity [11], warming [8], as well as disturbances [3]. Thus, an integrative assessment is essential for a comprehensive understanding of the responses and the underlying mechanisms of BSCs to anthropogenically caused climate change and intensified disturbances [5,6].

Sand burial is one of the most common disturbances that BSCs encounter in arid sandy desert ecosystems due to their surface niche and low stature [12,13,14,15]. Even when the burial depth is shallow, induced by wind or light animal activities, the coverage of BSCs is much more drastically reduced than that of vascular plants [16]. The prescribed climatic warming and aridity as well as the intensified anthropogenic disturbance predict that the threats of sand burial to BSCs would be more popular and influential [7,17]. Sand burial results in mechanical compression and changes the thermal, hydrological, aerobic and nutritious conditions of BSCs’ habitat [14,16,18,19]. Consequently, sand burial can affect the physiological activities, growth rate, the survivor, and some key ecological functions of the BSCs [15,19,20,21]. In the longer-term run, sand burial may act as a filter that determines the survival and succession sequence of BSCs (named after their external-photoautotrophs, e.g., moss and lichens) [6,16,20], but how sand burial affects the internal-heterotrophs (e.g., bacteria, micro fungi) inhabiting BSCs remains poorly understood. Considering that microbially driven organ sedimentary topsoil assemblages known as biological soil crusts (BSCs) normally exist as a thin mantle that only penetrates the upper centimeters of soil or less, their impact on the surrounding ecosystem is extraordinary.

In the Tengger desert, the fourth largest desert in China, sand burial produces multiple organic horizons of “fossilized BSCs” in areas where BSCs have survived burial stress and barren spaces where they have not [16]. These burial events macroscopically determine the succession sequence of BSCs [22]. Previously, we have demonstrated that the effects of sand burial on greenhouse gas emissions and effects of the carbon and nitrogen cycle are not exclusively by the alteration of water and sand burial temperature environmental factors to explain that crust sand burial changed in a species, especially the influence of the composition on the diversity of the inhibition effect of climate change on the system function of the microbial community [19,20]. Meanwhile, recent studies exhibited clear shifts in soil microbial community structure after desert revegetation in the same area [4,23]. In some sense, sand burial means desertification or reversal of revegetation. Therefore, a series of attractive questions are raised: (1) does sand burial influence microbial communities inhabiting BSCs? and if thus, (2) whether sand burial imposes negative effects on microbial diversity; (3) whether the changes in microbial communities caused by sand burial correlated with the alteration in soil chemical properties? In addition, was fungi harder to recover than bacteria [4]?

In this work, we study microbial communities that harbor BSCs at two successive stages in the vegetation of the Shapotou desert. Because this site hosts well-defined sequences of BSCs and sand burial perturbations at various depths, it provides an ideal region to study the response of microbial communities inhabiting BSCs to sand burial. We hypothesized that (1) sand burial changes the abundance and composition of the bacterial and fungal communities in the later-succession; (2) sand burial has a greater effect on bacterial community composition than that on fungal community; (3) sand burial influences bacterial and fungal communities by modifying the soil chemical properties affecting BSCs. To test these hypotheses, we studied the bacterial and fungal communities within BSCs that had been buried by different depths (unburied, shallow burial, and deep burial) of sand and were at the two adjacent successional stages (cyanobacterial crust for early succession stage and mixed crusts for the late succession stage). This study can improve our understanding of the effect of sand burial on BSCs in arid sandy desert ecosystems and the associated mechanisms underlying it.

## 2. Materials and Methods

### 2.1. Study Area

The study was conducted at Shapotou (37°32′–37°26′ N, 105°02′–104°30′ E), on the southeast fringe of the Tengger Desert within an altitude range of 1300-1350 m above sea level (a.s.l.). This area is a typical ecotone between desert steppe and steppified desert, and is a transitional belt between oasis and desert environments (Figure S1).

According to the meteorological station of Shapotou, the annual mean temperature from 1955 to 2016 was 10 °C and the annual mean precipitation was 186 mm, about 80% of which occurred between May and September [24]. The mean annual wind velocity is 2.9 m/s, the mean annual potential evaporation is 2900 mm, and the mean annual number of days with dust storms is 59. The soil is loose, infertile, and mobile and can thus be classified as orthic sierozem and Aeolian sandy soil [25]. The non-crusted dunes consist of 99.7% sand and 0.3% fine particles (silt and clay), whereas BSCs contain about 35% fine particles [25]. A non-irrigated vegetation system was initially established in 1956 on mobile dune sands (with no BSC cover) to protect the Baotou-Lanzhou railway line from sand burial. This was subsequently extended in 1964, 1981, and 1987 using the same method. BSCs begin to colonize the dune surface following burial stress relief and surface stabilization. Over the past 60 years, BSCs have sequentially experienced three typical successional stages (i.e., cyanobacteria crust, mixed crust, and moss crust). In this region, the dominant cyanobacteria species are *Microcoleus vaginatus* Gom., *Lyngbya cryptovaginatus* Schk., *Scytonema javanicum* Kütz.; the dominant moss species are *Bryum argenteum* Hedw., *Didymodo nvinealis* (Brid.) Zand., and *Syntrichia caninervis* Mitt; and the dominant lichen species are *Collema cocophorum* Tuck. and *Endocarpon pusillum* Hedw [26]. While the wind-blown sand burial stress on the growth of BSCs gradually decreases each year, these crusts are inevitably exposed to repeated sand or dust burial to various depths. This burial is caused by animal activity (ant, lizard, and rabbit burrows) and wind blowing [20,27], both of which generate different burial depths, where 0.5- and 10-mm depths are the most common and represent shallow burial (BSCs are partially buried and easily recover) and deep burial (BSCs are completely buried and it is difficult for them to recover), respectively.

### 2.2. Sand Burial Treatment and Sampling

Two typical BSC successional stages, cyanobacterial crust and mixed crust (lichen, green algae, cyanobacteria), were selected without replication on two windward slopes of the areas where revegetation had started in 1987 (the earlier successional BSC stage) and 1956 (the latter two BSC successional stages), respectively, to minimize the disturbance on the highly-protected revegetation system (Figure S1). Even though we selected nine small-size samples for each BSC stage and thus their samples for each BSC stage and burial depth, which seemed to be pseudo-replication, these samples were planned to ensure that their spatial interdependencies were minimized.

Firstly, cylindrical polyvinyl chloride (PVC) tubes (104 mm diameter, 200 mm depth) were randomly placed in the soil covered by the two successional BSC stages in late March 2013. A total of 9 tubes were used to measure each BSC succession stage. They were then randomly divided into two groups and subjected to three sand burial depth treatments. A total of 0 (control) and 65 or 130 g of air-dried drifting sand was distributed gently and evenly over the samples to simulate three depths of sand (0, 0.5, and 10 mm, respectively) (Figure S2). The uppermost edges of the PVC tubes were kept 0.5 cm above the lower inner flat surface to prevent the sand from being blown away. Sampling was conducted in September 2014. To avoid edge effects, all samples were taken from the middle of the tubes using sterilized cylindrical PVC tubes that were 5 cm in diameter. For each successional stage, the BSC samples that had been subjected to the same burial depth treatment were mixed and then immediately transported to the laboratory and stored at −80 °C until the nucleic acids were extracted. When the extraction was finished, all samples were then air-dried, crushed, and passed through a 2-mm sieve in the laboratory for chemical properties analysis.

### 2.3. Soil Physical and Chemical Properties

The chemical properties were determined according to Li et al. (2006) [25]. The pH of a suspension of soil-water at a ratio of 1:5 was determined using a calibrated pH meter (PHS-4, Jiangsu Manufactory of Electrical Analysis Instruments, Jiangyin, China). Total organic carbon (*TOC*) was measured using the K_2_Cr_2_O_7_ methods described by the Agriculture Chemistry Specialty Council, Soil Science Society of China (1983). Total nitrogen (*TN*) was measured using a Kjeldahl analysis system (*Kjeltec* 8400, Foss, Hillerød, Denmark), and available nitrogen (*AN*) was determined by the alkaline diffusion method. Total phosphorus (*TP*) was determined by colorimetry using sulfuric acid-perchloric acid digestion, and available phosphorus (*AP*) was determined colorimetrically by the molybdenum blue method after extraction with sodium bicarbonate solution.

### 2.4. Soil DNA Extraction and High-Throughput Sequencing

Microbial DNA was extracted from the BSC samples using an E.Z.N.A. DNA Kit (Omega Bio-Tek, Norcross, GA, USA) according to the manufacturer’s protocols. The combined nine DNA extracts (three replicates for each BSC) were then used for the subsequent polymerase chain reaction (PCR) and sequencing analyses. The bacterial 16S ribosomal RNA gene and the fungal ITS rRNA genes were amplified by PCR (95 °C for 3 min, followed by 25 cycles at 95 °C for 30 s, 55 °C for 30 s, and 72 °C for 45 s, with a final extension at 72 °C for 10 min) using primers 338F and 806R [28], and ITS1F and 2043R [29], respectively. The PCR reactions were performed in triplicate using a 20 μL mixture containing 2 μL of 5 × FastPfu buffer, 2 μL of 2.5 mM dNTPs, 0.8 μL of each primer (5 μM), 0.2 μL of FastPfu polymerase, and 10 ng of template DNA.

Amplicons were extracted from 2% agarose gels and purified using an AxyPrepDNA gel extraction kit (Axygen Biosciences, Union City, CA, USA) according to the manufacturer’s instructions. They were then quantified using QuantiFluor-ST fluorometer (Promega, Madison, WI, USA). Purified amplicons were pooled in equimolar quantities and paired-end sequenced (2 × 300) on an Illumina (San Diego, CA, USA) MiSeq platform PE300. The raw reads were deposited into the NCBI Sequence Read Archive (SRA) database (Accession Number: SRP113398 and PRJNA887223).

### 2.5. Bioinformatics and Statistical Analysis

The paired-end reads from the original DNA fragments were merged using FLASH software [30], which is designed to merge paired-end reads when there are overlaps between reads 1 and 2. Paired-end reads were assigned to each sample according to their unique barcodes. The raw sequencing data were quality-filtered using the following criteria: (i) the reads were truncated at any site that received an average quality score <20 over a 50-bp sliding window, and truncated reads that were shorter than 50 bp were removed; and (ii) any reads that had exact barcode matches or two nucleotide mismatches during primer matching and contained ambiguous characters were discarded. Only sequences with overlaps longer than 10 bp were assembled according to their overlap sequence. Reads that could not be assembled were discarded. Operational taxonomic units (OTUs) were clustered with a 97% similarity cutoff using UPARSE [31] and chimeric sequences were identified and removed using UCHIME [32]. Singleton OTUs was removed. The taxonomies of the 16S and ITS rRNA were identified using the RDP Classifier (http://rdp.cme.msu.edu/ accessed on 25 September 2021) against SILVA (v. 132) database (https://www.arb-silva.de/ accessed on 25 September 2021) or Unite (v. 8.0) (https://unite.ut.ee/ accessed on 25 September 2021) [33], respectively, a confidence threshold of 0.7. All samples were normalized at the same sequence depth. QIIME 1.8.0 software [34] and the OTUs were then used to calculate the alpha-diversity and beta-diversity metrics. The relationships among the microbial communities in the different samples were analyzed using nonmetric multidimensional scaling (NMDS), and the linear discriminant analysis (LDA) effect size (LEfSe) to identify the significantly different bacterial and fungal groups in the various burial groups [35]. The relevance of soil chemical properties (i.e., *TN*, *TP*, *TK*, *AK*, *AP*, *TO,* and *pH*) and their ability to explain the distribution patterns of the microbial communities in the different BSC samples was analyzed by distance-based redundancy analysis (db-RDA) and the Monte Carlo permutation test.

## 3. Results and Discussions

Traditional, culture-dependent methods were commonly utilized to isolate and identify the members in the microbial communities, but a vast majority of environmental microorganisms (≥99%) cannot be cultured. Therefore, traditional research methods commonly underestimate the soil microbial diversity, and cannot real truly reflect the microbial community composition in-depth and detail. In this study, we adopted an innovative molecular strategy based on Illumina MiSeq sequencing to analyze the bacterial and fungal communities within BSCs that had been buried at the three depths (unburied, shallow burial, and deep burial) of sand burial and were at the two adjacent successional stages (cyanobacterial crust for early succession stage and mixed crusts for the late succession stage).

### 3.1. Microbial Diversity and Community Composition of BSCs and Feedback on Sand Burial

We obtained a total of 452,107 valid sequences that were classified into 56,595 bacterial OTUs from the 18 samples (Table S2). The samples contained from 16,541 to 47,085 sequences, with the number of OTUs ranging from 3760 to 10,187. For the fungi (Table S3), we obtained 628,760 valid sequences and 4276 OTUs. Each library contained from 27,030 to 45,054 valid sequences, with the number of OTUs ranging from 411 to 1065.

**Figure 1:**
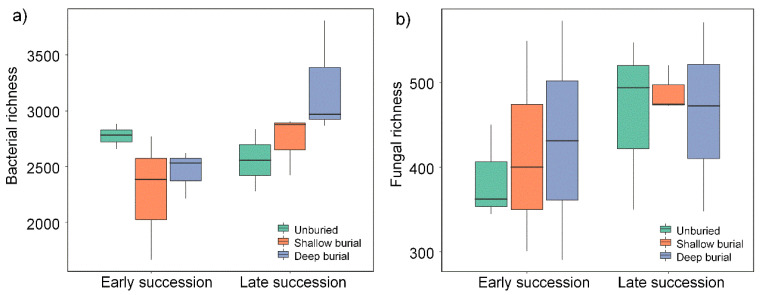
Richness of bacterial communities (**a**) and fungal communities (**b**) in the three soil depths under early and late succession. There are no significant effects of succession periods or sample depths on bacterial or fungal richness.

**Figure 2:**
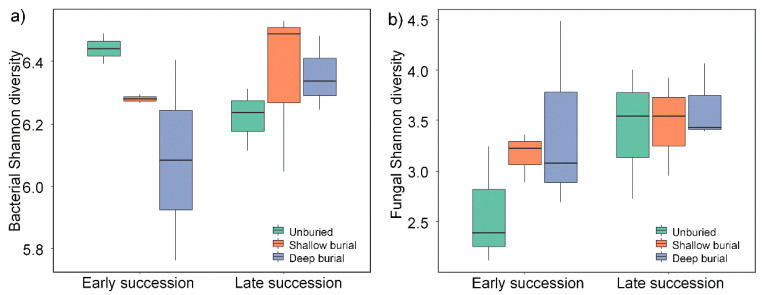
Shannon diversity of bacterial communities (**a**) and fungal communities (**b**) in the three soil depths under early and late succession. There are no significant effects of succession periods or sample depths on bacterial or fungal Shannon diversity.

The α-diversity was estimated based on the number of OTUs, Chao 1, and ACE, and the Shannon and Simpson indices. Tables S1 and S2 show the results for the bacteria and fungi, respectively. Community species richness (Chao 1 index) and community diversity (Shannon index) for the six groups were shown in Figure 1 and Figure 2, respectively. For the bacteria, the Chao1 index and the Shannon index ranged from 2726.08 to 5069.64 and from 5.978 to 9.420, respectively. For the fungi, the Chao1 index and the Shannon index ranged from 203 to 501 and from 3.02 to 6.44, respectively. Clearly, both the indexes of Chao 1 and Shannon for the bacterial community were much larger than those for the fungal communities. Additionally, for the bacteria, the mean value of the Chao1 index and Shannon index ranged from 2273 ± 322 (SBES) to 3216 ± 297 (DBLS) and from 6.08 ± 0.18 (DBES) to 6.44 ± 0.03 (UBES), respectively (Table S4). For the fungi, the mean value of the Chao1 index and Shannon index ranged from 386 ± 33 (UBES) to 489 ± 16 (SBLS) and from 2.58 ± 0.34 (UBES) to 3.63 ± 0.22 (DBLS), respectively (Table S4). This may be attributed to the fact that sand burial reduces solar radiation intensity, reduces water evaporation and increases soil moisture [15], thus providing a more suitable living microenvironment for bacteria. Overall, it is implied that sandy soil burial significantly affected the bacterial diversity in the soil crust, but had little effect on the fungal diversity. In the late stage of sand burial, the average richness of bacterial species in soil crust was higher than that in the early stage, but not evident. However, different burial depths did not significantly alter microbial species richness and Shannon diversity.

**Figure 3:**
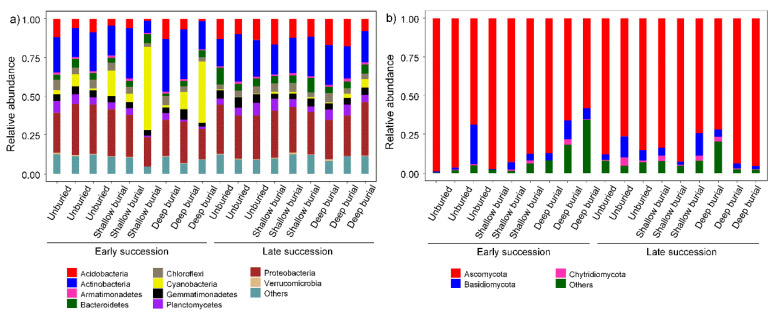
The community composition of bacteria (**a**) and fungi (**b**) at the phylum level in the present study.

**Figure 4:**
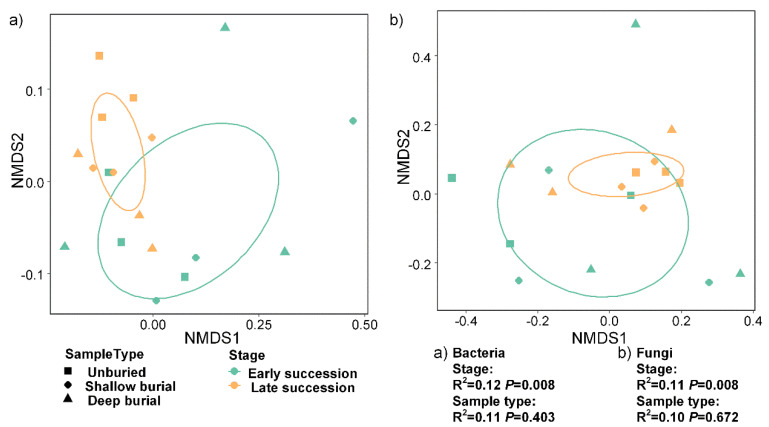
NMDS ordination of the bacterial (**a**) and fungal (**b**) communities.

Ten most dominant bacterial phyla across all 18 samples from six groups were *Proteobacteria* (23.0–30.2%), *Actinobacteria* (20.2–28.2%), *Cyanobacteria* (0.4–25.3%), *Acidobacteria* (3.8–14.1%), *Bacteriodetes* (3.3–6.7%), *Planctomycetota* (3.1–6.1%), *Gemmatimonadota* (3.9–5.8%), *Chloroflexi* (3.8–5.6%), *Armatimonadetes* (1.0–1.4%), and *Verrucomicrobiota* (0.3–0.8%) (Figure 3a, Table S5). For the fungal community, *Ascomycota* (70.3–92.6%) was the most dominant phylum across all 18 samples from six groups, followed by *Basidiomycota* (3.10–9.10%) and *Chytridiomycota* (0.4–2.4%) (Figure 3b, Table S6). The NMDS analysis based on the Bray-Curtis distance was used to infer the β-diversity patterning of soil bacterial and fungal community composition (Figure 4), and the results showed that the bacterial and fungal community composition was significantly correlated to the sand burial stage (Bacteria: R^2^ = 0.12, *p* = 0.008; fungi: R^2^ = 0.11, *p* = 0.008), while different burial depths had a weak effect on the microbial community’s composition of BSCs (Bacteria: R^2^ = 0.11, *p* = 0.403; fungi: R^2^ = 0.11, *p* = 0.008). Previous studies showed that the *Cyanobacteria*, *Proteobacteria*, and *Actinobacteria* were the three most dominant bacterial phyla of BSCs in desert areas, including the Sonoran desert (North America) [36,37], Gurbantunggut desert (West China) [38], and Colorado plateau [39]. In this study, four bacterial phyla of the *Proteobacteria*, *Actinobacteria*, *Cyanobacteria*, and *Acidobacteria* were common in BSCs from the Shapotou desert revegetation areas. This result is in agreement with previous study in this region [40]. Additionally, the predominance of *Ascomycota* in all samples confirmed that it is the dominant fungal phylum in the Shapotou desert region. Previously, the effects of sand burial on external photoautotrophs of BSCs are reported to vary with the thickness and the timing of burial, as well as crustal development stages [15,16,19]. Shallow sand burial promotes biocrust growth, while thicker sand burial reduces the PSII photochemical efficiency, chlorophyll a, and extracellular polysaccharide content of biocrust, and leads to biocrystal cryptogam death [21] after long-term, deep sand burial. This study found similar trends that sand burial effects on microbial communities were mediated by successional stages of BSCs and highly relied on burial depth.

### 3.2. Identification of Indicator Taxa and then Used Them to Indicate the Succession Stage of the Crust in the Same Environment

**Figure 5:**
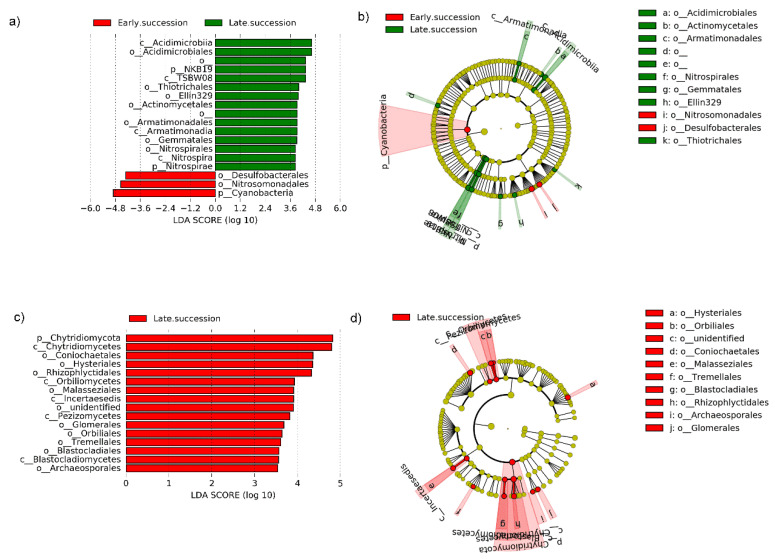
Cladogram indicates the phylogenetic distribution of microbial lineages under different succession periods, and each circle’s diameter is proportional to the given taxon’s relative abundance. Different colors represent different treatments and dominant taxonomy groups. (**a**) Indicator bacteria with linear discriminant analysis (LDA) scores of 2 or greater in bacterial communities under different succession periods. (**b**) Phylogenetic distribution of bacterial lineages under different succession periods. (**c**) Indicator fungi with LDA scores of 2 or greater in fungal communities under different succession periods. (**d**) Phylogenetic distribution of fungal lineages under different succession periods.

**Figure 6:**
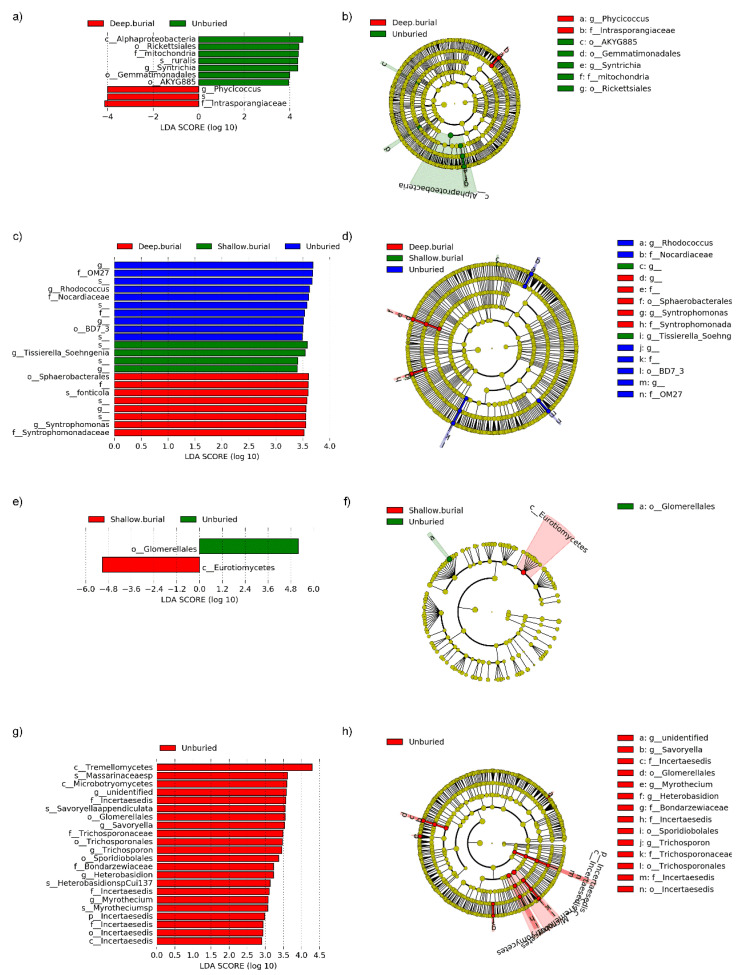
Cladogram indicates the phylogenetic distribution of microbial lineages under different succession periods, and each circle’s diameter is proportional to the given taxon’s relative abundance. Different colors represent different treatments and dominant taxonomy groups. (**a**) Indicator bacteria with linear discriminant analysis (LDA) scores of 2 or greater in bacterial communities under different soil depths during early succession. (**b**) Phylogenetic distribution of bacterial lineages under different soil depths during early succession. (**c**) Indicator bacteria with LDA scores of 2 or greater in bacterial communities under different soil depths during late succession. (**d**) Phylogenetic distribution of bacterial lineages under different soil depths during late succession. (**e**) Indicator fungi with LDA scores of 2 or greater in fungal communities under different soil depths during early succession. (**f**) Phylogenetic distribution of fungal lineages under different soil depths during early succession. (**g**) Indicator fungi with LDA scores of 2 or greater in fungal communities under different soil depths during late succession. (**h**) Phylogenetic distribution of fungal lineages under different soil depths during late succession.

We applied linear discriminant analysis (LDA) to explore the microbial species significantly enriched under different burial depths and succession stages (Figure 5 and Figure 6).

Firstly, only 3 bacterial taxonomic groups (*Cyanobacteria*, *Nitrosomonadales*, and *Desulfobacterota*) were enriched at the early succession stage (Figure 5a,b), while 15 bacterial taxonomic groups (*Acidimicrobiia*, *Acidimicrobiales*, NKB19, TSBW08, Thiotrichales, Ellin329, *Actinomycetales*, *Armatimonadales*, *Armatimonadia*, *Gemmatales*, *Nitrospirales*, *Nitrospira*, and *Nitrospirae*) are enriched at the late succession stage (Figure 5a,b). Unlike bacteria, a total of 16 fungal taxonomic groups (*Chytridiomycota*, *Chytridiomycetes*, *Coniochaetales*, *Hysteriales*, *Rhizophlyctidales*, *Orbiliomycetes*, *Malasseziales*, Incertaesedis, unidentified, *Pezizomycetes*, *Glomerales*, *Orbiliales*, *Tremellales*, *Blastocladiales*, *Blastocladiomycetes*, and *Archaeosporales*) enriched in the late successional stage, and these species were mainly from the *Ascomycota* (Figure 5c,d). These results support previous findings that microbial communities in late successional soil crusts have relatively higher species richness. Furthermore, we explored which microbial species were significantly enriched at different sand burial depths (Figure 6). Firstly, we compared the difference of indicator species between unburied and deep burial at the early succession stage (Figure 6a,b), the results showed that 7 bacterial taxonomic groups (*Alpha-Proteobacteria*, *Rickettsiales*, *Mitochondria*, *Ruralis*, *Syntrichia*, *Gemmatimonadales*, and AKYG885) were enriched in the unburied samples, while only 3 bacterial taxonomic groups (mainly *Phycicoccus* and *Intrasporangiaeae*) were enriched in deep burial samples. For the fungi, the results showed that only 1 fungal taxonomic group was enriched in unburied samples (Glomerellales) and the shallow burial samples (Eurotiomycetes) at the early succession stage, respectively (Figure 6e,f). Subsequently, at the late succession stage, 10, 4, and 8 bacterial taxonomic groups were enriched in the unburied, shallow burial, and deep burial samples, respectively (Figure 6c,d). Unlike bacteria, more than 20 fungal taxonomic groups were enriched in the unburied samples (Figure 6g,h).

By comparing indicator species in BSCs before and after sand burial, we find that the bacterial and fungal community composition of BSCs changes significantly. For example, *Cyanobacteria* was the one of most abundant bacteria phyla in the BSCs at the early succession stage, but disappeared at the late succession stage (Figure 5a,b). On the one hand, although *Cyanobacteria* can survive for a long time in arid environments, the increased humidity caused by sand burial during drought may enhance the decomposition of bacteria by regulating their respiratory and metabolic processes, thus leading to the degradation of exopolysaccharides. On the other hand, cyanobacterial cells are also prone to autolysis due to lack of sufficient light to activate photosynthesis [41]. The combined effects of the two aspects eventually reduced the relative abundance of *Cyanobacteria* in the whole bacterial community. Meanwhile, several bacterial groups detected only at the late succession stage, such as *Nitrospirales*, *Nitrospira*, and *Nitrospirae*, they could promote nitrite (NO_2_^−^) and subsequently nitrate (NO_3_^−^). That implied that the nitrogen cycle intensity increased significantly at the late succussion stage. Overall, there are significant differences in the bacterial and fungal community compositions of the BSCs at the two succussion stages of the different sand burial depth. Additionally, several indicator taxa were enriched in the different stages or in the different sand-buried stages.

### 3.3. Possible Mechanisms Underlying the Effects of Sand Burial on Bacterial and Fungal Communities Inhabiting Biological Soil Crusts

**Figure 7:**
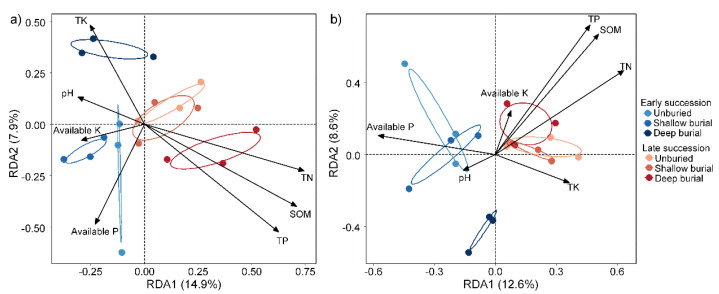
Distance-based redundancy analysis (RDA) of the relationship between the environmental factors and (**a**) bacterial and (**b**) fungal communities.

We applied distance-based redundancy analysis (db-RDA) to explore which environmental factors influence microbial community composition of the BSCs. (Figure 7). For the bacteria, *TN* (R^2^ = 0.43, *p* = 0.01), *SOM* (R^2^ = 0.40, *p* = 0.021), and *TP* (R^2^ = 0.37, *p* = 0.01) had great influence on the bacterial community composition of BSCs, while soil *pH* and *TK* had little influence on it. Likewise, *TN* (R^2^ = 0.43, *p* = 0.01) also contributed the most to the changes in fungal community composition of the BSCs, followed by *SOM* (R^2^ = 0.42, *p* = 0.016) and *TP* (R^2^ = 0.39, *p* = 0.029), which significantly affected fungal community compositions. Previously, it has been demonstrated that variations in edaphic factors (e.g., soil moisture and soil texture) could affect microbial communities by altering diffusion and adsorption of available substrates. For example, soil pH has marked effects on microbial biomass, community structure, and response to substrate addition, and that there was a higher proportion of fungal biomass in lower pH soil [42]. Bacterial richness and diversity increased as water potential decreased and the soil became drier, but they were not affected by texture [43]. Additionally, microclimatic differences may have a comparable effect on micro fungi in BSCs and sand burial changed the substrate quality [44] and environmental conditions for soil fungi (from a comparatively rich organic crust layer to a poor mineral sandy layer) [27]. Previous studies have reported that sand burial leads to changes in environmental conditions, such as increasing the bioavailability of nitrogen [14], reducing the extracellular polysaccharides content [33] and changing the dark respiration rate [16] in the BSCs.

## 4. Conclusions

In summary, the bacterial diversity and richness of BSCs is affected by sand burial compared to the unburied treatment, but the effect on fungal diversity is modest. The average abundance of bacteria in the BSCs at the late stages of sand burial is higher than that at the early stages, but not significantly. At the same time, the microbial richness and Shannon diversity of the BSCs do not change significantly at different burial depths. Additionally, several different bacterial and fungal taxa were identified at the early succession stages (e.g., *Cyanobacteria*) and late succession stages (e.g., *Nitrospirales*, *Nitrospira*, and *Nitrospirae*) of the BSCs, respectively. These taxa of microorganisms could be considered indicator species and used to reflect the stage of sand burial succession. In addition, changes in soil properties caused by sand burial have been suggested as a possible cause of changes in the bacterial and fungal community composition in BSCs. These results suggest that sand burial may drive the succession of microbial communities inhabiting BSCs in deforested regions and similar regions around the world.

## Data Availability

The raw data of microbial sequencing were deposited into the NCBI database (accession numbers: Sequence Read Archive: SRP113398 and PRJNA887223).

## Supplementary Materials

The following supporting information can be downloaded at: https://www.mdpi.com/article/10.3390/microorganisms10102010/s1, Figure S1: Diagram showing the location and main landscape of the revegetated area in the Tengger Desert; Figure S2: Experimental plots covered by cyanobacterial crusts and mixed crusts, with tubes for control and for simulation of shallow sand burial, and deep sand burial; Table S1: Sampling information; Table S2: Phylotype coverage and diversity estimation of the 16S rRNA gene libraries of the samples from the MiSeq sequencing analysis; Table S3: Phylotype coverage and diversity estimation of the ITS rRNA gene libraries of the samples from the MiSeq sequencing analysis; Table S4: The richness and Shannon diversity of bacteria and fungi; Table S5: Relative abundance of bacterial phyla; Table S6: Relative abundance of fungal phyla.
