# Supplementary material for: Differential Influences of Wind-Blown Sand Burial on Bacterial and Fungal Communities Inhabiting Biological Soil Crusts in a Temperate Desert, China
Source: Microorganisms. 2022 Oct 11;10(10):2010. doi: 10.3390/microorganisms10102010 (PMC9609826; doi:10.3390/microorganisms10102010)
Supplement: Supplementary file 1 [file microorganisms-10-02010-s001.zip › microorganisms-1951737-supplementary.pdf]

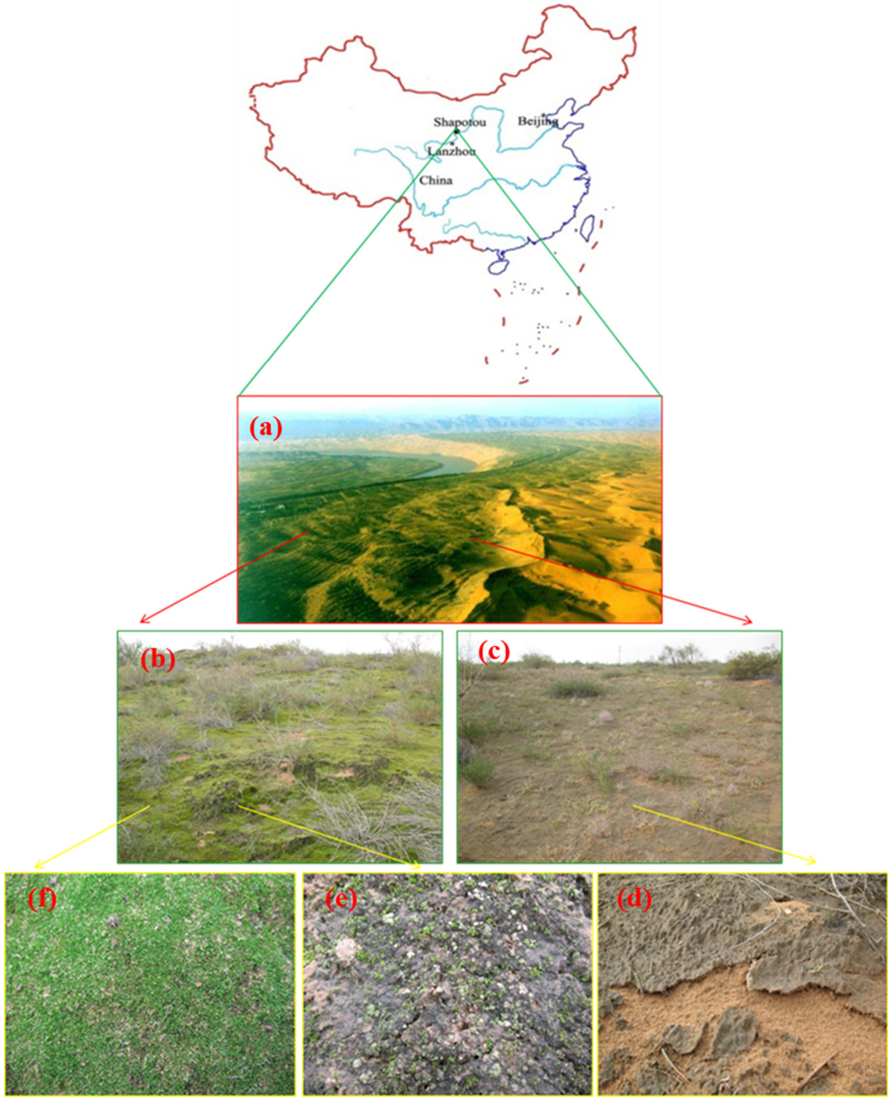

Figure S1. Diagram showing the location and main landscape of the revegetated area in the Tengger Desert **(a).** The revegetated area established in 1956 (**b**) and 1987 (c); cyanobacterial crust (**d**), mixed crust (**e**), and moss crust (Bryum argenteum Hedw) (**f**).

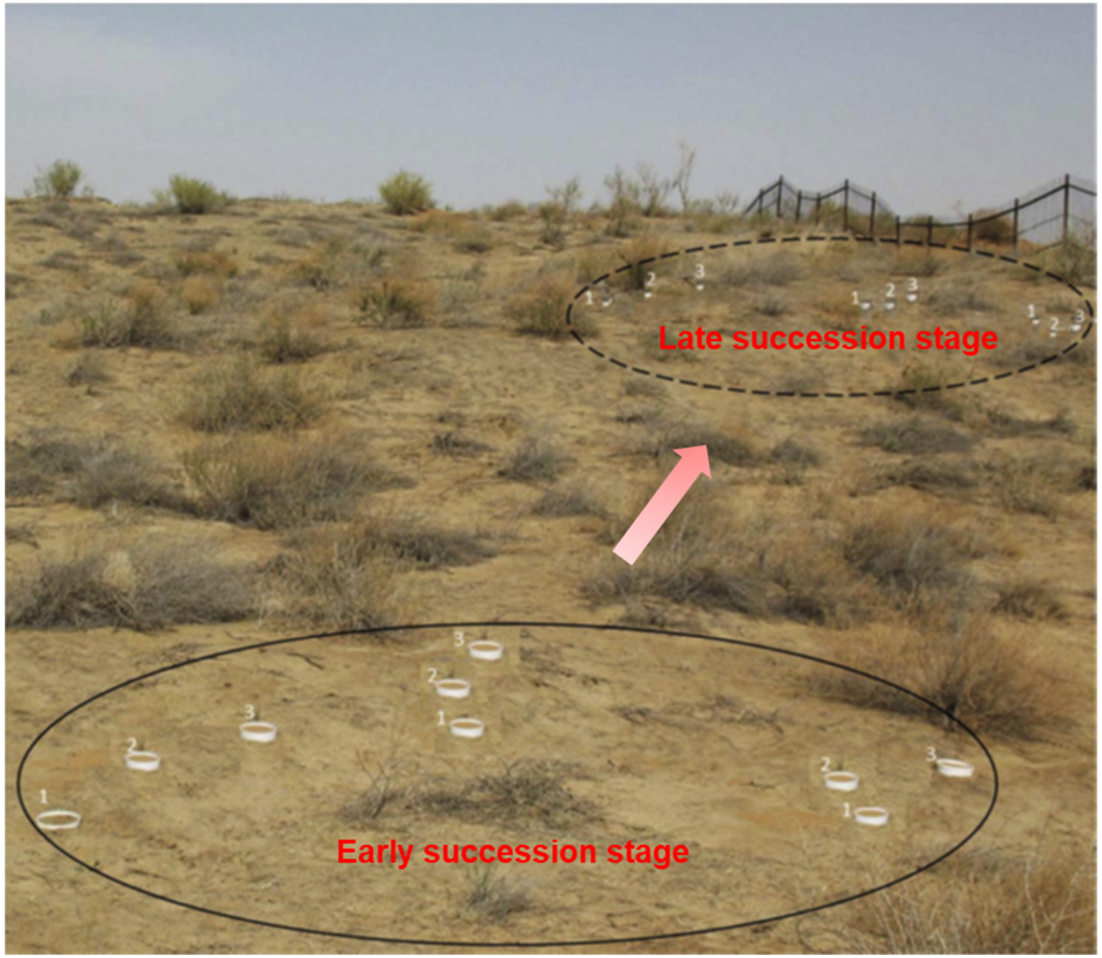

Figure S2. Experimental plots covered by cyanobacterial crusts (Early successional stage, surrounded by solid line) and mixed crusts (Late successional stage, surrounded by dashed line), with tubes for control (**1**) and for simulation of shallow sand burial (**2**), and deep (**3**) sand burial.

Table S1. Sampling information.

Sample ID	Crusts Type	Sand Buried Depth	The Succession Stage
**56MC**	Cyanobacteria	Unburied	Early succession
**81WLC**	Cyanobacteria	Unburied	Early succession
**87WBC**	Cyanobacteria	Unburied	Early succession
**87WAC**	Cyanobacteria	Shallow burial	Early succession
**BMC1**	Cyanobacteria	Shallow burial	Early succession
**87CC**	Cyanobacteria	Shallow burial	Early succession
**BMC2**	Cyanobacteria	Deep burial	Early succession
**87BCC**	Cyanobacteria	Deep burial	Early succession
**87LAC**	Cyanobacteria	Deep burial	Early succession
**56BC**	Mixed crusts	Unburied	Late succession
**56DC**	Mixed crusts	Unburied	Late succession
**56RC**	Mixed crusts	Unburied	Late succession
**BBC1**	Mixed crusts	Shallow burial	Late succession
**81WBC**	Mixed crusts	Shallow burial	Late succession
**SD**	Mixed crusts	Shallow burial	Late succession
**BBC2**	Mixed crusts	Deep burial	Late succession
**SB**	Mixed crusts	Deep burial	Late succession
**SA**	Mixed crusts	Deep burial	Late succession

Table S2. Phylotype coverage and diversity estimation of the 16S rRNA gene libraries of the samples from the MiSeq sequencing analysis.

Sample ID	Chao1	ACE	Simpson	Shannon	Valid Reads	OTUs
**56MC**	4386.702	4686.355	0.994597	9.361533	20927	6118
**81WLC**	4378.041	4445.103	0.995101	9.224376	22799	6042
**87WBC**	4516.646	4825.59	0.993945	9.291807	22870	6055
**87WAC**	4378.42	4597.006	0.994002	9.079645	25839	6401
**BMC1**	4069.557	4280.438	0.992001	9.040551	16541	4932
**87CC**	2726.079	2692.988	0.905593	5.977513	29483	3760
**BMC2**	3879.248	3999.714	0.994265	9.239113	22231	6574
**87BCC**	3620.27	3659.278	0.983984	8.315238	29261	6273
**87LAC**	3517.008	3598.112	0.983244	7.764043	29660	5318
**56BC**	3751.399	3906.027	0.990909	8.822142	18487	4433
**56DC**	4505.202	4658.206	0.99262	8.996463	25836	6745
**56RC**	4153.783	4333.582	0.993739	9.104841	20649	5677
**BBC1**	4482.739	4606.666	0.99466	9.419937	23651	6881
**81WBC**	4395.877	4750.961	0.994507	9.359793	24253	6525
**SD**	4062.716	4257.966	0.989457	8.723465	18863	4918
**BBC2**	4303.38	4517.515	0.995009	9.349704	27942	7640
**SB**	5069.64	5296.543	0.986335	9.011065	47085	10187
**SA**	4339.065	4625.086	0.993432	9.142462	25730	6770

Table S3. Phylotype coverage and diversity estimation of the ITS rRNA gene libraries of the samples from the MiSeq sequencing analysis .

Sample ID	Chao1	ACE	Simpson	Shannon	Valid Reads	OTUs
**56MC**	229	263.32223	0.7081338	3.4028053	33352	538
**81WLC**	263	297.93212	0.5891974	3.0207654	37669	478
**87WBC**	350	381.34773	0.906425	4.6408207	33662	683
**87WAC**	203	262.99297	0.9303671	4.6144531	29740	411
**BMC1**	279	318.52553	0.8989066	4.8068444	37217	533
**87CC**	491	515.9974	0.6918262	4.154832	36795	839
**BMC2**	245	272.96108	0.8573335	3.8693982	45054	432
**87BCC**	488	518.76165	0.9637265	6.437987	30033	1065
**87LAC**	301	352.02201	0.870866	4.3967402	33965	652
**56BC**	275	300.21562	0.7875493	3.9051473	30103	448
**56DC**	413	433.83847	0.9502822	5.7457804	33256	663
**56RC**	461	497.22747	0.9127812	5.0883395	38650	790
**BBC1**	433	460.72968	0.9541202	5.6288382	39503	705
**81WBC**	351	406.54883	0.8442732	4.2200655	37036	620
**SD**	393	393	0.8936205	5.0794049	27030	717
**BBC2**	501	521.98476	0.9520074	5.8456654	39957	842
**SB**	361	387.04134	0.9149917	4.8562339	33014	655
**SA**	242	278.8612	0.9333843	4.9100543	32724	450

Table S4. The richness and Shannon diversity of bacteria and fungi.

	UBES	SBES	DBES	UBLS	SBLS	DBLS
Bacterial Shannon	6.44 ± 0.03a	6.28 ± 0.01a	6.08 ± 0.18a	6.22 ± 0.06a	6.35 ± 0.15a	6.35 ± 0.07a
Bacterial richness	2773 ± 64ab	2273 ± 322b	2455 ± 123b	2558 ± 160ab	2733 ± 155ab	3216 ± 297a
Fungal Shannon	2.58 ± 0.34a	3.16 ± 0.14a	3.42 ± 0.54a	3.42 ± 0.37a	3.47 ± 0.28a	3.63 ± 0.22a
Fugal richness	386 ± 33a	417 ± 72a	432 ± 81a	464 ± 59a	489 ± 16a	464 ± 65a

Note: UBES: Unburied at the early succession stage; SBES: Shallow burial at the early succession stage, DBES: Deep burial at the early succession stage; UBLS: Unburied at the late succession stage; SBLS: Shallow burial at the late succession stage; DBLS: Deep burial at the late succession stage. Different lowercase letters (a and b) indicate significant differences ($P < 0.05$) among the groups.

Table S5. Relative abundance of bacterial phyla.

Phyla	UBES	SBES	DBES	UBLS	SBLS	DBLS
Acidobacteria	0.088 ± 0.017ab	0.038 ± 0.015b	0.070 ± 0.034ab	0.121 ± 0.012a	0.135 ± 0.015a	0.141 ± 0.032a
Actinobacteria	0.221 ± 0.019a	0.202 ± 0.070a	0.282 ± 0.050a	0.242 ± 0.038a	0.226 ± 0.018a	0.224 ± 0.018a
Armatimona-detes	0.014 ± 0.002a	0.011 ± 0.003a	0.010 ± 0.002a	0.012 ± 0.002a	0.010 ± 0.001a	0.014 ± 0.002a
Bacteriodetes	0.044 ± 0.007a	0.042 ± 0.008a	0.033 ± 0.009a	0.067 ± 0.021a	0.065 ± 0.015a	0.049 ± 0.005a
Chloroflexi	0.056 ± 0.008a	0.038 ± 0.008a	0.040 ± 0.010a	0.040 ± 0.005a	0.047 ± 0.008a	0.044 ± 0.009a
Cyanobacteria	0.038 ± 0.020a	0.253 ± 0.147a	0.174 ± 0.115a	0.004 ± 0.001a	0.010 ± 0.001a	0.029 ± 0.014a
Gemmati-monadota	0.047 ± 0.003a	0.039 ± 0.001a	0.044 ± 0.012a	0.058 ± 0.004a	0.041 ± 0.005a	0.043 ± 0.002a
Planctomyce-tota	0.061 ± 0.009a	0.031 ± 0.012ab	0.024 ± 0.010b	0.056 ± 0.013ab	0.054 ± 0.011ab	0.061 ± 0.007a
Proteobacteria	0.302 ± 0.023a	0.256 ± 0.033a	0.230 ± 0.020a	0.293 ± 0.013a	0.291 ± 0.008a	0.287 ± 0.029a
Verrucomicrobi-ota	0.008 ± 0.002a	0.003 ± 0.001a	0.004 ± 0.002a	0.005 ± 0.000a	0.006 ± 0.002a	0.006 ± 0.002a
Other	0.121 ± 0.005a	0.086 ± 0.021a	0.089 ± 0.012a	0.101 ± 0.011a	0.115 ± 0.009a	0.103 ± 0.010a

Note: UBES: Unburied at the early succession stage; SBES: Shallow burial at the early succession stage, DBES: Deep burial at the early succession stage; UBLS: Unburied at the late succession stage; SBLS: Shallow burial at the late succession stage; DBLS: Deep burial at the late succession stage. Different lowercase letters (a and b) indicate significant differences ($P < 0.05$) among the groups.

Table S6. Relative abundance of fungal phyla.

Phyla	UBES	SBES	DBES	UBLS	SBLS	DBLS
Ascomycota	0.880 ± 0.097a	0.926 ± 0.027a	0.703 ± 0.087a	0.831 ± 0.035a	0.835 ± 0.052a	0.871 ± 0.075a
Basidiomycota	0.091 ± 0.082a	0.031 ± 0.013a	0.080 ± 0.022a	0.079 ± 0.030a	0.073 ± 0.038a	0.033 ± 0.006a
Chytridiomycota	0.004 ± 0.002a	0.009 ± 0.005a	0.014 ± 0.011a	0.024 ± 0.014a	0.024 ± 0.009a	0.011 ± 0.009a
Other	0.025 ± 0.013b	0.033 ± 0.016b	0.204 ± 0.076a	0.066 ± 0.009b	0.069 ± 0.010b	0.085 ± 0.061ab

Note: UBES: Unburied at the early succession stage; SBES: Shallow burial at the early succession stage, DBES: Deep burial at the early succession stage; UBLS: Unburied at the late succession stage; SBLS: Shallow burial at the late succession stage; DBLS: Deep burial at the late succession stage. Different lowercase letters (a and b) indicate significant differences ($P < 0.05$) among the groups.
